# Theoretical Thermodynamic Efficiency Limit of Isothermal Solar Fuel Generation from H_2_O/CO_2_ Splitting in Membrane Reactors

**DOI:** 10.3390/molecules26227047

**Published:** 2021-11-22

**Authors:** Hongsheng Wang, Hui Kong, Jian Wang, Mingkai Liu, Bosheng Su, Sean-Thomas B. Lundin

**Affiliations:** 1 MOE Key Laboratory of Hydrodynamic Machinery Transients, School of Power and Mechanical Engineering, Wuhan University, Wuhan 430072, China; 2 Department of Chemical System Engineering, School of Engineering, The University of Tokyo, 7-3-1 Hongo, Bunkyo-ku, Tokyo 113-8656, Japan; 3 School of Mechanical Engineering, Beijing Institute of Technology, Beijing 100081, China; konghui2020@bit.edu.cn; 4 School of Energy and Environment, City University of Hong Kong, Hong Kong, China; jian.wang@cityu.edu.hk; 5 Institute of Engineering Thermophysics, Chinese Academy of Sciences, 11 Beisihuanxi Rd., Beijing 100190, China; liumingkai@iet.cn; 6 College of Marine Equipment and Mechanical Engineering, Jimei University, Xiamen 361021, China; 7 Fujian Province Key Laboratory of Energy Cleaning Utilization and Development, Xiamen 361021, China

**Keywords:** solar fuel, hydrogen generation, CO_2_ splitting, H_2_O splitting, CO generation, membrane reactor

## Abstract

Solar fuel generation from thermochemical H_2_O or CO_2_ splitting is a promising and attractive approach for harvesting fuel without CO_2_ emissions. Yet, low conversion and high reaction temperature restrict its application. One method of increasing conversion at a lower temperature is to implement oxygen permeable membranes (OPM) into a membrane reactor configuration. This allows for the selective separation of generated oxygen and causes a forward shift in the equilibrium of H_2_O or CO_2_ splitting reactions. In this research, solar-driven fuel production via H_2_O or CO_2_ splitting with an OPM reactor is modeled in isothermal operation, with an emphasis on the calculation of the theoretical thermodynamic efficiency of the system. In addition to the energy required for the high temperature of the reaction, the energy required for maintaining low oxygen permeate pressure for oxygen removal has a large influence on the overall thermodynamic efficiency. The theoretical first-law thermodynamic efficiency is calculated using separation exergy, an electrochemical O_2_ pump, and a vacuum pump, which shows a maximum efficiency of 63.8%, 61.7%, and 8.00% for H_2_O splitting, respectively, and 63.6%, 61.5%, and 16.7% for CO_2_ splitting, respectively, in a temperature range of 800 **°**C to 2000 °C. The theoretical second-law thermodynamic efficiency is 55.7% and 65.7% for both H_2_O splitting and CO_2_ splitting at 2000 °C. An efficient O_2_ separation method is extremely crucial to achieve high thermodynamic efficiency, especially in the separation efficiency range of 0–20% and in relatively low reaction temperatures. This research is also applicable in other isothermal H_2_O or CO_2_ splitting systems (e.g., chemical cycling) due to similar thermodynamics.

## 1. Introduction

The world faces environmental issues (e.g., global warming, haze) induced by large amounts of fossil fuel utilization that produce large quantities of CO_x_, NO_x_, SO_x_, and ash emissions. Instead of fossil energy, clean and renewable energy, such as abundant solar energy, should be used to partially address these issues [1,2]. While solar irradiation has low energy density and is discontinuous in time and space, concentrating solar energy for solar fuels generation is considered a promising approach to efficiently generate transportable and long-term storable energy carriers with high energy density [3,4,5]. To decrease the extremely high reaction temperature (c.a. 4000 °C for ΔG = 0 in H_2_O splitting reaction), isothermal solar H_2_O or CO_2_ splitting is usually carried out in membrane reactor [6,7,8,9,10] or chemical cycle systems [11,12,13,14,15,16], both of which require a low permeate oxygen partial pressure to separate oxygen and shift the equilibrium toward high conversion at relatively low temperature. As they are thermodynamically similar, this research focuses on H_2_O or CO_2_ splitting in oxygen-permeable membrane (OPM) reactors. OPM reactors have the advantage of being one-step operations, which allows for continuous utilization of solar energy and offers a promising route for high-efficiency solar energy harvesting [7,17]. Among solar fuels, H_2_ and CO are attractive due to their high heat values as well as the abundance of their source chemicals, H_2_O and CO_2_. The equations for H_2_ and CO generation from H_2_O and CO_2_ are:

(1) $$H_{2}O\to H_{2}+{0.5O}_{2}$$

(2) $${\mathrm{CO}}_{2}\to \mathrm{CO}+{0.5O}_{2}$$

A selective barrier for H_2_O splitting, which uses the chemical potential difference of the species to transport a product across the barrier, was first proposed independently by two research groups [10,18]. This developed into the study of thermochemical reactions using membrane reactors. Wang et al. [9] proposed an isothermal OPM reactor for H_2_O splitting driven by solar energy, which can separate oxygen from the reaction continuously and shift the equilibrium of conversion. Zhu et al. [19] proposed ceria as a candidate material of the OPM reactor for solar fuel production and analyzed the thermodynamic and kinetic performance of the OPM reactor. Li et al. [20] studied the effects of gas heat recovery and reactor flow configurations on thermodynamic performance, and maximum efficiencies of 1.3% and 3.2% were obtained in H_2_O and CO_2_ splitting, respectively. Steinfeld and his collaborators [6,7] established the high-temperature solar thermochemical membrane reactor, and experimentally validated stable H_2_ and CO production by OPM (CeO_2_ membrane) reactor. The CO generation rate is 0.024 μmol s^−1^ cm^−2^ at 1600 °C and 3 × 10^−6^ bar with 3500 suns radiation [7]. Abanades and his collaborators utilized mixed ionic-electronic conducting (MIEC), which is made of CeO_2_, for solar thermochemical CO_2_ splitting, and a CO production rate of 0.071 μmol s^−1^ cm^−2^ at 1550 °C was achieved [21]. Then they further optimize the OPM as composite membranes with CeO_2_ and perovskite materials, which achieved a CO production rate of 0.13 μmol s^−1^ cm^−2^ at 1550 °C with a CO_2_ flow rate of 1 L min^−1^ and sweep gas (Ar, 99.9999%) flow rate of 1 L min^−1^ between the two sides of the membrane [22]. Recent research related to thermochemical CO_2_ splitting with MIEC membrane has been reviewed by Wu and Ghoniem [8]. The membrane reactor for solar fuel generation has been regarded as a single-step process, and has the inherent advantage of being environmentally friendly, energetically, and economically efficient, apart from demonstrating synthetic competency and elegance [23]. However, the low experimental energy efficiency (less than 1% [7,22]) still limits the utilization of membrane reactors.

To analyze and optimize the energy efficiency of H_2_O or CO_2_ splitting in a membrane reactor, the solar thermochemical H_2_O or CO_2_ splitting process is simulated in an OPM reactor by Aspen Plus V8.2, which can conveniently integrate with other simulations (e.g., power generation or chemical engineering) for the comprehensive study of solar energy conversion and storage processes. The energy efficiency variation is systematically discussed under various temperatures and pressures, and the thermodynamic performance limits for H_2_ or CO generation are calculated and discussed.

## 2. System Description and Theoretical Formulation

### 2.1. System Description

**Figure 1:**
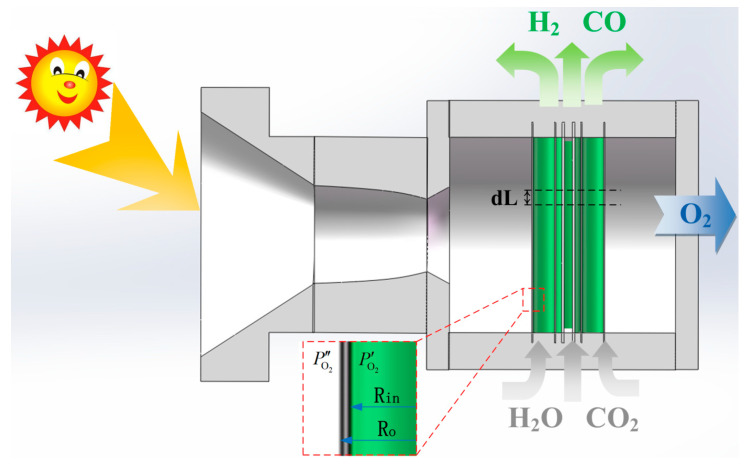
Illustration of conceptual OPM reactor.

A conceptual solar thermochemical OPM reactor is illustrated in Figure 1, in which a bundle of OPM tubes made of dense MIEC ceramic material is exposed to concentrated sunlight that heats the reactor to a temperature sufficiently high for both noticeable partial thermolysis of water or carbon dioxide and oxygen transport across the membrane. Point-focusing solar collector, (e.g., parabolic dish collector capable of reaching 1527 °C [3]), can be used for converting solar energy into thermal energy to drive the endothermic chemical reaction.

H_2_O or CO_2_ fed to the reactor is split within MIEC tubes, which allows the generated O_2_ to be separated via the imposed chemical potential difference between the two sides of the membrane. The O_2_ removal shifts the equilibrium of the reaction toward higher conversion at lower reaction temperatures compared to a non-membrane integrated system. The chemical potential difference can be maintained by vacuum pumping or feeding sweep gas, both of which have the same separation exergy in thermodynamics. The dimensions of the reactor are outlined in Table 1, where *R*_o_ and *R*_in_ are the outer and inner radius of the membrane tube, respectively, and *L* is the length of the membrane tubes. For later computations, d*L* is the length of control volumes into which *L* is divided.

### 2.2. MIEC Membrane Kinetics

Oxygen transport through a dense MIEC membrane involves several steps [24,25]: (1) O_2_ adsorbs and dissociates into oxygen ions on the surface; (2) charged species and electron holes diffuse through the bulk; (3) oxygen ions recombine on the permeate side surface and the generated oxygen molecules desorb. The local oxygen permeation flux for a tubular reactor that takes these steps into account can be expressed by [26]:

(3) $$J_{O_{2}}=\frac{K_{r}D_{v}\left[{\left(P_{O_{2}}^{\prime \prime }\right)}^{0.5}-{\left(P_{O_{2}}^{\prime \prime }\right)}^{0.5}\right]}{2\left(R_{o}-R_{\mathrm{in}}\right)K_{f}\left(P_{O_{2}}^{\prime }P_{O_{2}}^{\prime \prime }\right)+\frac{R_{m}}{R_{\mathrm{in}}}D_{v}{\left(P_{O_{2}}^{\prime }\right)}^{0.5}+\frac{R_{m}}{R_{o}}D_{v}{\left(P_{O_{2}}^{\prime \prime }\right)}^{0.5}}$$

where $P_{O_{2}}^{\prime }$ and $P_{O_{2}}^{\prime \prime }$ are the partial pressures of oxygen across the membrane corresponding to the high- and low-pressure sides (feeding side and permeate side), respectively. The logarithmic mean radius is defined as $R_{m}=\left(R_{o}-R_{\mathrm{in}}\right)/\ln\left(R_{o}/R_{\mathrm{in}}\right)$, which is used to calculate the membrane area (2π*R*_m_*L*); *D_v_* is the diffusion coefficient of oxygen vacancies in the membrane; and *K_f_* and *K_r_* are the forward and backward reaction rates of the oxygen dissociation/association reactions described above [27]:

(4) $$\mathrm{Oxygen}\;\mathrm{dissociation}:\frac{1}{2}O_{2}+V_{Ö}\overset{K_{f}/K_{r}}{⇌}O_{O}^{\times }+2\dot{h}$$

(5) $$\mathrm{Oxygen}\;\mathrm{association}:O_{O}^{\times }+2\dot{h}\overset{K_{r}/K_{f}}{⇌}\frac{1}{2}O_{2}+V_{Ö}$$

where $V_{Ö}$, $O_{O}^{\times }$, and $\dot{h}$ are oxygen vacancy, oxygen ion, and electron-hole. For this work, La_0.6_Sr_0.4_Co_0.8_Fe_0.2_O_3−δ_ was chosen as a candidate material, with associated data for *K_f_*, *K_r_*, and *D_v_* [28]. It should be noted that the thermodynamic results (final conversion and thermodynamic efficiency) are only influenced by the reaction temperature, reaction pressure, and permeate pressure, and it is not affected by the OPM material properties, meaning the thermodynamic results in this research are suitable for any OPM material.

To represent the characteristics of OPM reactor, the following assumptions have been adopted: (1) steady-state operation of the membrane reactor [29]; (2) ideal plug-flow operation [30]; (3) conformation of gases to the ideal state equation [31]; (4) constant total pressure on each side of the membrane [32]. The ideal plug-flow operation can be assumed for flow conditions with a Péctlet number above 100 [33], and in this work, the Péclet number is calculated to be above 5200 for H_2_O splitting and above 44,000 for CO_2_ splitting. Additionally, for the conditions in this study, deviation from ideal behavior for all gases is expected to be less than 1%.

**Figure 2:**
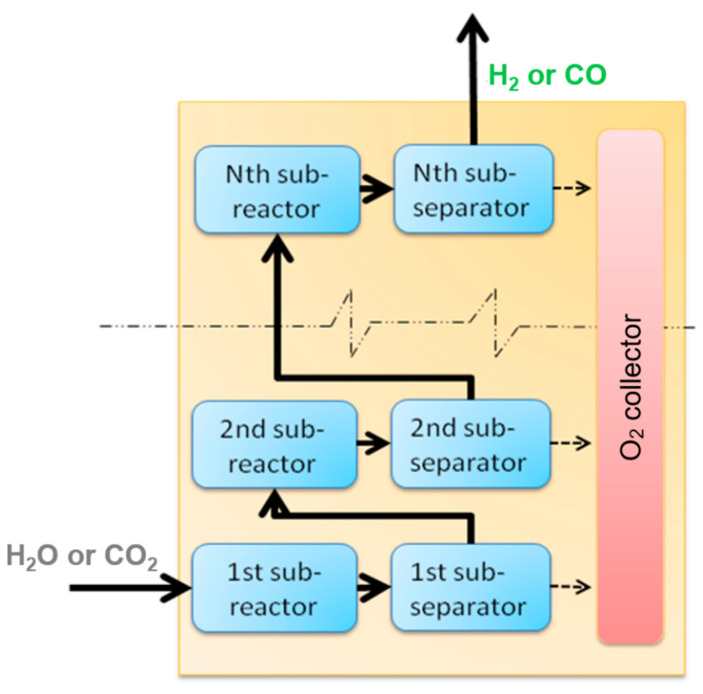
Sequential modular simulation diagram of OPM reactor.

In the simulation, the OPM reactor is divided into several control volumes, each of which contains a sub-reactor and a sub-separator. The reactant gas reacts in the sub-reactor to generate oxygen and hydrogen or carbon monoxide via the calculation of Gibbs free energy minimization and then flows into a sub-separator to separate the oxygen generated. The processes are repeated several times until the reactant gas flows out the membrane tube. The process of simulation is expressed in Figure 2.

### 2.3. Thermodynamic Performance

Based on the calculation of the amount of H_2_ or CO generation, thermodynamic performance can be further analyzed to give insight into the energy conversion process. Herein, the first-law thermodynamic efficiency (energy efficiency) and the second-law thermodynamic efficiency (exergy efficiency) are selected as two indicators to gauge thermodynamic performance. The first-law thermodynamic efficiency is the ratio of chemical energy contained in the output product to the system energy input, defined as [34]:

(6) $$\eta_{\mathrm{HHV}}=\frac{n_{\mathrm{fuel}}\cdot HHV_{\mathrm{fuel}}}{\eta_{\mathrm{abs}}^{-1}\cdot (Q_{\mathrm{preheat}}+Q_{\mathrm{enthalpy}})+W_{p}}$$

where *n*_fuel_ denotes the molar amount of the fuel generated (H_2_ or CO); *HHV*_fuel_ is the higher heating value of the generated fuel, which is taken as 286 kJ mol^−1^ and 283 kJ mol^−1^ for H_2_ and CO, respectively; *η*_abs_ is the absorption efficiency of the collector, expressed as [10,32]:

(7) $$\eta_{\mathrm{abs}}=\alpha \eta_{\mathrm{opt}}^{}-\frac{\varepsilon \cdot \sigma \cdot {\left(T_{H}+273.15\right)}^{4}}{DNI\cdot C_{\mathrm{collector}}}$$

where *α* and *ε* are the absorptivity and emissivity of the collector, both taken as 1 for the blackbody radiation model [10]; *η*_opt_ is the optical efficiency of the solar parabolic dish collector, taken as 85% [35]; *σ* is the Stefan-Boltzmann’s constant; *T*_H_ is the temperature (unit: °C) of the collector; *DNI* is the direct normal irradiation, taken as 1000 W m^−2^ [36]; *C*_collector_ is the concentration ratio of the collector, set as 5000 [10] for the dish collector in this research. *Q*_preheat_ is the heat consumed to raise the temperatures of carbon dioxide and water from room temperature to reaction temperature, and calculated as:

(8) $$Q_{\mathrm{preheat}}=\left\{ \begin{array}{l} n_{H_{2}O}\cdot (\int_{T_{L}}^{100\;{}^{\circ}C}C_{{P,H}_{2}O}dT+40.872+\int_{100\;{}^{\circ}C}^{T_{H}}C_{{P,H}_{2}O}dT),{\;(H}_{2}O\;\mathrm{splitting})\;\\ n_{{\mathrm{CO}}_{2}}\cdot \int_{T_{L}}^{T_{H}}C_{{P,\mathrm{CO}}_{2}}dT,{\;(\mathrm{CO}}_{2}\;\mathrm{splitting}) \end{array}\right.$$

where *n*_H2O_ and *n*_CO2_ are the molar amounts of H_2_O and CO_2_ reacted; *C*_p,H2O,_ and *C*_p,CO2_ are the specific heat capacities of H_2_O and CO_2_; 40.872 kJ mol^−1^ is the latent heat of water evaporation. *Q*_enthalpy_ is the heat consumed by the enthalpy change of H_2_O or CO_2_ splitting reaction; *W*_P_ is the energy consumed to maintain a low pressure for oxygen separation and expressed as:

(9) $$W_{P}=n_{O_{2}}\cdot RT_{0}\ln\left(P_{0}/P_{O_{2}}^{\prime \prime }\right)/\eta_{p}$$

where *n*_O2,out_ is the molar amount of separated O_2_; *R* is the universal gas constant, taken as 8.314 J mol^−1^ K^−1^; *T*_0_ is room temperature, given as 298.15 K; *P*_0_ is the ambient pressure, fixed at 1 bar; *P*_O2,out_ is the permeate pressure. *η*_P_ is the separation efficiency, defined as the ratio of separation exergy output to separation energy input. For the upper limit of the thermodynamic efficiency, the separation exergy will be taken for calculation (*η*_P_ = 100%); for an electrochemical oxygen pump, the thermodynamics are similar to a solid oxide electrolysis cell, both of which mainly have activation overpotential, concentration overpotential, and ohmic overpotential, and the separation efficiency is calculated as the electrolytic efficiency here, which is taken as 80% [37]; for a vacuum pump, the separation efficiency is the mechanical efficiency of the vacuum pump, which can be calculated as [38]:

(10) $$\eta_{p}={\left(\frac{P_{O_{2}}^{\prime \prime }}{P^{⊖}}\right)}^{0.544}$$

where *P*^⊖^ is the standard pressure. To further analyze the exergy conversion process, the exergy efficiency is defined as [39]:

(11) $$\eta_{\mathrm{ex}}=\frac{n_{\mathrm{fuel}}\cdot Ex_{\mathrm{fuel}}}{Ex_{\mathrm{solar}}+W_{p}},$$

(12) $$Ex_{\mathrm{solar}}=\left(1-\frac{4T_{0}}{3T_{\mathrm{sun}}}+\frac{1}{3}\cdot {\left(\frac{T_{0}}{T_{\mathrm{sun}}}\right)}^{4}\right)\left(\eta_{\mathrm{abs}}^{-1}\cdot (Q_{\mathrm{preheat}}+Q_{\mathrm{enthalpy}})\right)$$

where *Ex*_fuel_ is the chemical exergy of H_2_ or CO, taken as 235 kJ mol^−1^ and 275 kJ mol^−1^, respectively; *Ex*_solar_ is the input exergy of solar energy. *T*_sun_ is the surface temperature of the sun, given as 5800 K. *W*_P_ is calculated as separation exergy in this case. In the energy efficiency and exergy efficiency calculation, the recovery of thermal energy contained in the products is not taken into account.

## 3. Results and Discussion

**Figure 3:**
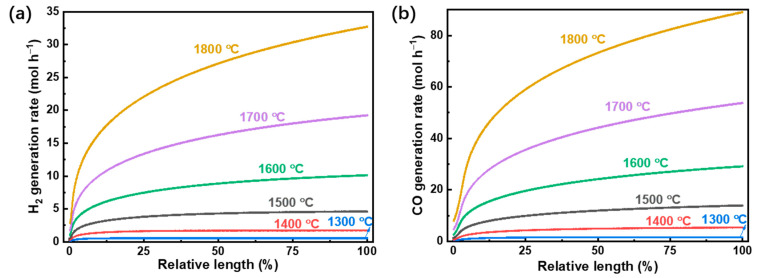
(**a**) H_2_ and (**b**) CO generation rate versus the relative length of the reactor.

The simulation was carried out with the model described above. The oxygen permeate pressure is fixed at 10^−5^ bar unless stated otherwise. The fuel gas generation rate and reactant conversion rate are calculated with a base case of reactant gas flow rate, which is 0.1 mol s^−1^. The H_2_ and CO generation rate was simulated in the software of Aspen Plus V8.2, and the results are given in Figure 3.

**Figure 4:**
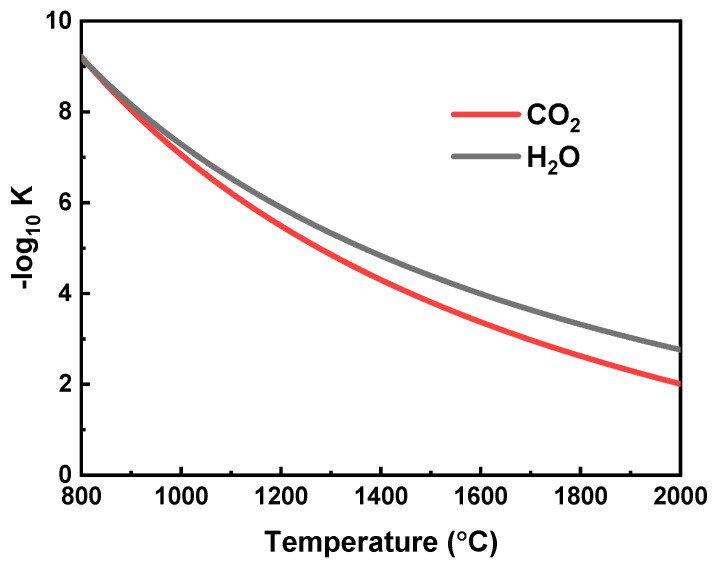
Thermodynamic equilibrium constants of H_2_O and CO_2_ [40].

As the temperature increases, the generation rates increase for both H_2_ and CO due to the reactions being endothermic. Notably, the CO generation rate is higher than that of H_2_ because the equilibrium constant of CO_2_ splitting is greater than that of H_2_O splitting for temperatures higher than 820 °C (Figure 4 [40]). At the maximum temperature calculated of 1800 °C and using a permeate pressure of 10^−5^ bar, the generation rates of H_2_ and CO are 32.7 mol h^−1^ and 89.1 mol h^−1^ (7.26 kmol h^−1^ m^−2^ (membrane area) and 19.8 kmol h^−1^ m^−2^), respectively.

**Figure 5:**
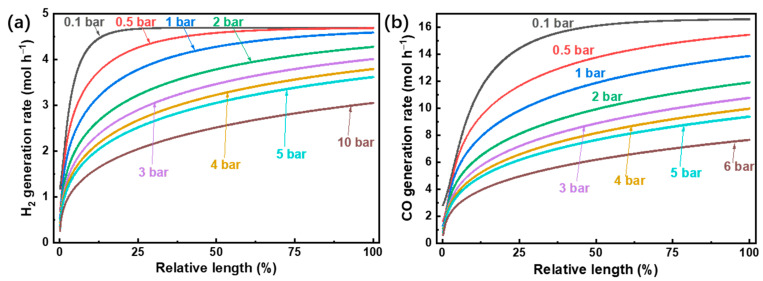
(**a**) H_2_ and (**b**) CO generation rate versus reaction pressure (T = 1500 °C).

**Figure 6:**
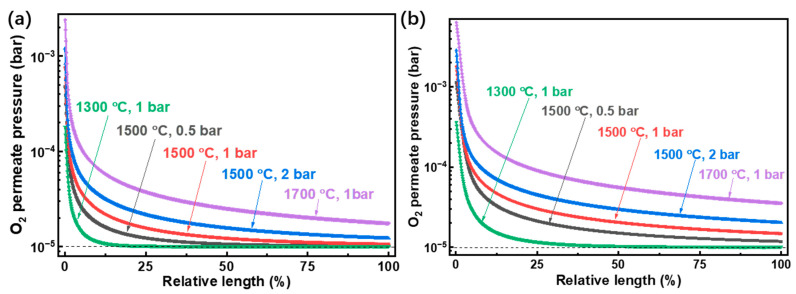
Influence of the (**a**) H_2_O and (**b**) CO_2_ splitting reaction temperature and pressure on O_2_ partial pressure difference between the two sides of the membrane.

Experimental analysis of thermodynamic performance has been performed at 1500 °C for similar systems [7,11,36], so the H_2_ and CO generation rates versus reaction pressure at 1500 °C are simulated and shown in Figure 5. As the reaction pressure increases, the fuel generation rate declines for both H_2_O and CO_2_ splitting. This is due to the mole increase for both H_2_O and CO_2_ splitting reactions, which results in volume expansion and a subsequent backward shift in equilibrium at higher reaction pressures. The generation rates of H_2_ and CO can reach 4.69 mol h^−1^ and 16.6 mol h^−1^ (1.04 kmol h^−1^ m^−2^ and 3.69 kmol h^−1^ m^−2^), respectively, at 1500 °C, a reaction pressure of 0.1 bar, and a permeate pressure of 10^−5^ bar. The fuel generation rates with low reaction pressure or low temperature reach stable conditions quickly, as the O_2_ partial pressure difference between the two sides of the membrane is smaller than that under high reaction pressure or temperature (given in Figure 6).

In Figure 6, the O_2_ partial pressure under equilibrium conditions in H_2_O (Figure 6a) and CO_2_ (Figure 6b) splitting reactions are given. Due to the difference in equilibrium constants of H_2_O and CO_2_ splitting as analyzed above, the O_2_ partial pressure in CO_2_ splitting is higher than that in H_2_O splitting under the same conditions. Also, higher temperatures and reaction pressures require longer tube lengths to achieve equilibrium conditions, where the O_2_ partial pressure equals the permeate pressure (10^−5^ bar).

**Figure 7:**
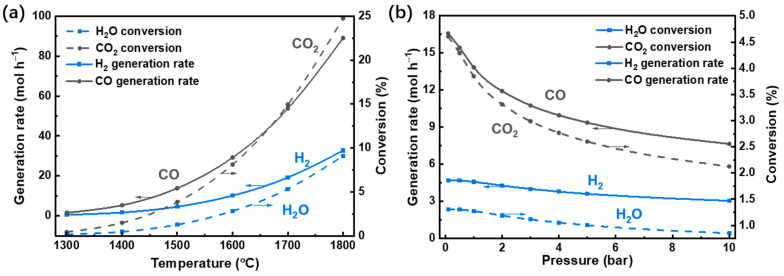
Generation rates of H_2_ and CO and conversions of H_2_O and CO_2_ various with (**a**) reaction temperature (under 1 bar) and (**b**) pressure (at 1500 °C).

The fuel gas generation rates and reactant conversion rates versus reaction temperature and pressure are shown in Figure 7. The reactant conversion directly influences the thermodynamic efficiencies, and the fuel generation rate reflects the potential for fuel generation amount in a certain time, both of which are significant indicators to gauge the system performance. When temperature increases, conversions, and generation rates increase due to the increase in equilibrium constants of Equations (1) and (2) (Figure 4), which indicates more fuel gas and oxygen will be generated. The trend of generation rates and conversions are similar because a high generation rate of fuel results in a high conversion of reactants. However, when the reaction pressure increases (Figure 7b), both conversion and generation rate decrease due to the reverse shift in equilibrium.

**Figure 8:**
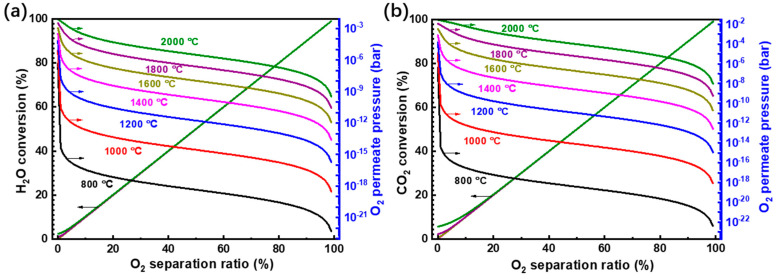
Conversion rates of (**a**) H_2_O and (**b**) CO_2_ versus O_2_ separation ratio and O_2_ permeate pressure.

To further analyze the thermodynamic performance of the system, the reaction temperature is extended from 800 °C to 2000 °C, which can be achieved by concentrated solar energy [41,42] and covers the common temperature range for high-temperature solar thermochemical reactions. The conversion and required O_2_ permeate pressure are exhibited in Figure 8. In Figure 8, the O_2_ separation ratio is defined as the ratio of the molar amount of O_2_ separated to half of the H_2_O fed into the reactor (the theoretical maximum O_2_ amount that can be separated). As the O_2_ separation ratio increases, the required O_2_ permeate pressure decreases and the conversions of both H_2_O and CO_2_ are enhanced. The O_2_ separation ratio has an almost linear relationship to H_2_O or CO_2_ conversion as the product generation amount is nearly twice that of the O_2_ separation amount, except for the simultaneous reaction at high temperature (e.g., 2000 °C) and low O_2_ separation ratio. Both high temperature and low permeate pressure are beneficial to high conversion rate, but the resultant increased requirement in energy to preheat the reactant and separate the O_2_ has a negative influence on the thermodynamic efficiency.

**Figure 9:**
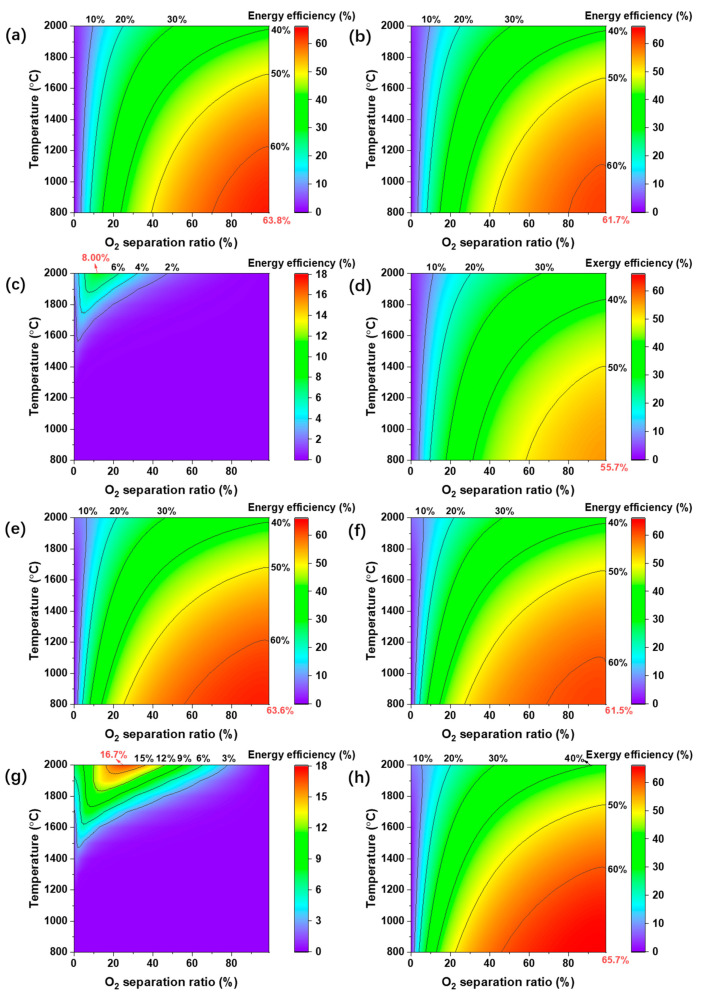
Thermodynamic efficiencies of high-temperature solar (**a**–**d**) H_2_O and (**e**–**h**) CO_2_ splitting reactions. The first-law thermodynamic efficiency is calculated with (**a**,**e**) separation exergy, (**b**,**f**) electrochemical oxygen pump, and (**c**,**g**) vacuum pump. Exergy efficiency is also analyzed for (**d**) H_2_O and (**h**) CO_2_ splitting. The energy efficiency ranges (color scale) are 0–66% in (**a**,**b**,**d**–**f**,**h**); and 0–18% in (**c**,**g**).

To further analyze the thermodynamic efficiency, the first-law thermodynamic efficiency with separation exergy, electrochemical O_2_ pump, and vacuum pump for O_2_ separation and the second-law thermodynamic efficiency are calculated and given in Figure 9. The first-law thermodynamic efficiency with separation exergy (Figure 9a,e), 80% separation efficiency (electrochemical O_2_ pump in Figure 9b,f), separation efficiency using Equation (10) (vacuum pump in Figure 9c,g), and the second-law thermodynamic efficiency (Figure 9d,h) of H_2_O and CO_2_ splitting are given for a temperature range of 800 °C to 2000 °C. Except Figure 9c,g, the other figures show an optimum efficiency at 800 °C with the highest O_2_ separation ratio (99%), mainly due to less radiation loss in low temperature and more fuel generation with a high O_2_ separation ratio. The optimum first-law thermodynamic efficiency is 63.8% and 63.6% for H_2_O splitting and CO_2_ splitting with separation exergy (Figure 9a,e), which denotes the upper bound of the first-law thermodynamic efficiency. The electrochemical O_2_ pump is driven by an electric potential difference between the two sides of the membrane, which has high energy efficiency. Based on an assumption of 80% separation efficiency, the optimum first-law thermodynamic efficiency is 61.7% and 61.5% for H_2_O splitting and CO_2_ splitting (Figure 9b,f). The energy efficiency of H_2_O splitting is slightly higher than that of CO_2_ splitting because the equilibrium constant of H_2_O splitting (6.53 × 10^−10^) is slightly higher than that of CO_2_ splitting (6.16 × 10^−10^) at 800 °C. This results in slightly higher conversion rates of H_2_O splitting compared to that of CO_2_ splitting.

**Figure 10:**
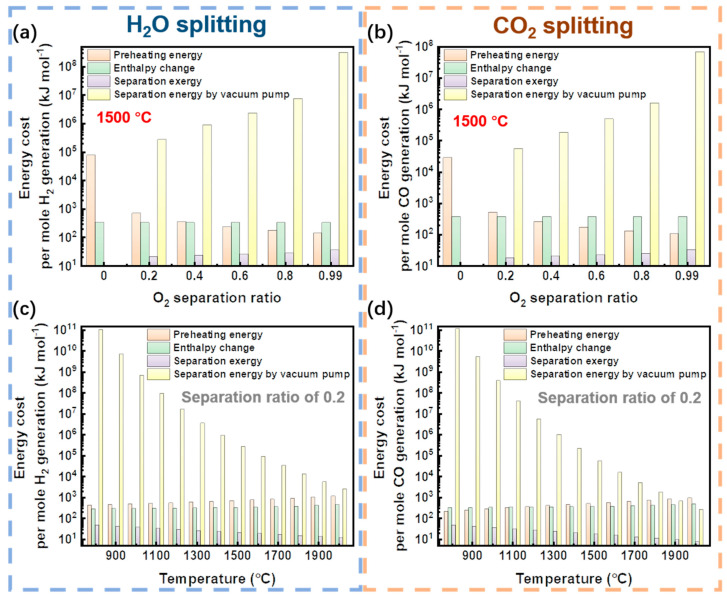
Energy cost per mole fuel generation in high-temperature solar thermochemical H_2_O or CO_2_ splitting reaction. (**a**,**b**) Energy cost versus O_2_ separation ratio; (**c**,**d**) Energy cost versus reaction temperature. (**a**,**b**) are at the reaction temperature of 1500 °C, and (**c**,**d**) are with the O_2_ separation ratio of 0.2. The thermal energy (preheating energy and enthalpy change) cost contains absorption efficiency (radiation loss and optical loss) given in Equation (7).

In Figure 9c,g, the optimum energy efficiency is 8.00% and 16.7% at 2000 °C with an O_2_ separation ratio of 12% (permeate pressure of 1.67 × 10^−4^ bar) and 25% (permeate pressure of 8.46 × 10^−4^ bar), respectively. As Equation (10) shows, the use of a vacuum pump results in very low efficiencies compared to the use of an electrochemical O_2_ pump, especially at low permeate pressures whereat the mechanical friction consumes significant amounts of energy [43] (exhibited in Figure 10). Rather than a vacuum pump, the permeate O_2_ pressure can be reduced by sweeping an inert gas, which allows the permeate total pressure to be atmospheric while maintaining a high O_2_ permeation driving force. However, this produces a mixed product stream of dilute O_2_ because the O_2_ partial pressure in the products is very low and must be even lower in the permeate stream. This results in a significant energy cost to recycle the O_2_, and the separation efficiency would be similar to that of using the vacuum pump. In a common high reaction temperature of 1500 °C, the theoretical first-law thermodynamic efficiency limits are only 1.45% (permeate pressure of 1.68 × 10^−5^ bar) and 3.50% (0.03 bar) for H_2_O splitting and CO_2_ splitting with O_2_ separation by vacuum pump, respectively, which conforms to the experimental results in the published literature [7,22]. Thus, exploring a high-efficient O_2_ separation method is very significant for approaching high energy efficiency. For the second-law thermodynamic efficiency calculation in Figure 9d,h, as CO exergy (275 kJ mol^−1^) is higher than that of H_2_ (235 kJ mol^−1^), the optimum efficiency of CO splitting is 65.7%, which is higher than that of H_2_O splitting (55.7%) at 800 °C. Based on the results in Figure 9, if O_2_ can be separated with high separation efficiency (e.g., electrochemical method), then a relatively low reaction temperature is better for the final thermodynamic efficiency of H_2_O or CO_2_ splitting. However, with traditional mechanical separation methods, a high reaction temperature is better to increase the permeate pressure and reach a high thermodynamic efficiency due to the low separation efficiencies.

To quantitively analyze the energy cost during these processes, the energy cost per mole fuel generation is calculated and expressed in Figure 10. Due to the energy cost listed here, the energy efficiency can be easily calculated, and the first-law thermodynamic efficiency can be calculated as:

(13) ηHHV=HHVfuelηabs−1⋅(qpreheatc+ΔH)+wp

where *q*_preheat_, Δ*H*, and *w*_p_ denote the thermal energy for preheating molar reactant, molar enthalpy change, and separation energy per mole H_2_ generation, respectively; *c* is the conversion, and $\eta_{\mathrm{abs}}^{-1}\cdot$*q*_preheat_/c, $\eta_{\mathrm{abs}}^{-1}\cdot$Δ*H*, and *w*_p_ are the preheating energy, energy for enthalpy change, and separation energy (separation exergy or separation energy by vacuum pump) in Figure 10.

In Figure 10a,b, the energy cost versus O_2_ separation ratio is exhibited for H_2_O and CO_2_ splitting reactions at a reaction temperature of 1500 °C. The separation ratio of 0 denotes the case without a membrane for O_2_ separation, which results in a low conversion rate that requires significantly more energy to preheat the necessarily increased quantity of reactants per mole of fuel generated. The preheating energy requirement decreases as the separation ratio increases, but the separation energy increases due to the requirement for a low permeate pressure. While the variation of separation exergy increases from 0 to 36.4 kJ (H_2_O splitting) or 33.1 kJ (CO_2_ splitting) for O_2_ separation from 0 to 99%, this increase is small compared to the preheating energy decrease. This means that the optimum efficiency with separation exergy increases with increasing O_2_ separation ratio.

**Figure 11:**
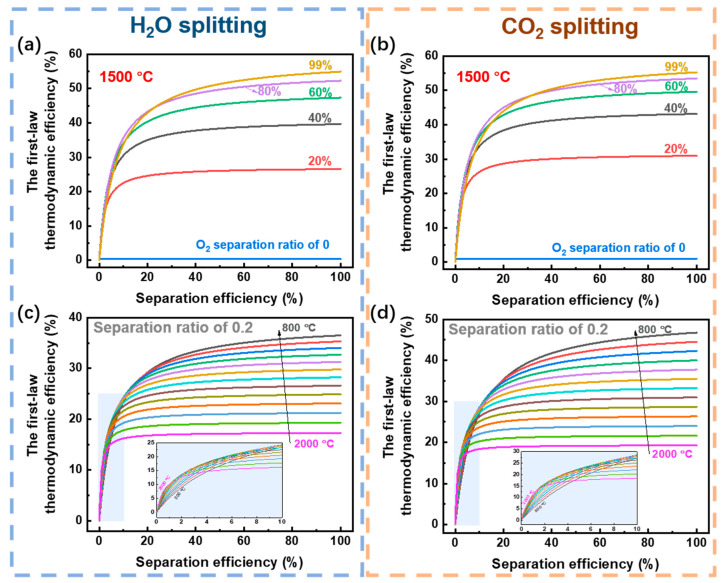
The first-law thermodynamic efficiency versus O_2_ separation efficiency. (**a**,**b**) are at a reaction temperature of 1500 °C, and (**c**,**d**) are with an O_2_ separation ratio of 0.2. The thermal energy (preheating energy and enthalpy change) cost contains the absorption efficiency (radiation loss and optical loss) given in Equation (7). The blue shaded regions in (**c**,**d**) are amplified to show more clearly the 0–10% separation efficiency range, and the results are given every 100 °C.

In Figure 10c,d, the energy cost versus reaction temperature with an O_2_ separation ratio of 0.2 is given. With increasing temperature, the radiation loss is increased and more preheating energy is required to maintain the same conversion rate (Figure 8a). As the permeate pressure declines, the separation energy increases due to the increased energy requirement of the separation method (herein the vacuum pump energy). In all cases, the enthalpy changes per mole fuel generated are almost constant, which only slightly varies with temperature. Thus, separation energy is the key parameter to influence the final energy efficiency. Although not discussed here, the energy cost for the second-law thermodynamic efficiency is similar to that of the first-law thermodynamic efficiency. To further demonstrate the significance of O_2_ separation efficiency, the influence of separation efficiency on the first-law thermodynamic efficiency of H_2_O and CO_2_ splitting is calculated in Figure 11.

As the separation efficiency increases, the first-law thermodynamic efficiency increases in all conditions. It should be noted that the separation efficiency range of 0–20%, has a great influence on the first-law thermodynamic efficiency, which indicates that it is very important to increase the separation efficiency within the low range. While, most of the physical separation method has a low separation efficiency under low permeate pressure, and to fulfill the separation efficiency 20%, the permeate pressure should be larger than 5.19 × 10^−2^ bar for a vacuum pump based on Equation (10). To reach this permeate pressure, a high reaction temperature is required (Figure 8), which has a negative influence on thermodynamic efficiency due to high preheating energy. Thus, a high-efficient oxygen separation method is needed to approach a high energy efficiency for H_2_ and CO generation from thermochemical H_2_O and CO_2_ splitting.

Though this research focuses on isothermal membrane reactors, the analyses and results in this research are also suitable for other isothermal H_2_O or CO_2_ splitting reactions (e.g., isothermal chemical cycle) due to the similarity in thermodynamics.

## 4. Conclusions

A theoretical framework was established for high-temperature solar thermochemical fuel production with oxygen-permeable membrane reactors. Theoretical thermodynamic efficiency limits of H_2_O and CO_2_ splitting reactions were analyzed and the primary conclusions include:

(1) O_2_ separation efficiency has the largest influence on the thermodynamic efficiency, especially in the separation efficiency range of 0–20%, where is the traditional physical separation method, which means that a highly efficient O_2_ separation method is crucial for high energy efficiency in membrane reactors for H_2_O or CO_2_ splitting;

(2) The theoretical first-law thermodynamic efficiency with separation exergy, electrochemical O_2_ pump, and vacuum pump can achieve 63.8%, 61.7%, and 8.00% for H_2_O splitting, and 63.6%, 61.5%, and 16.7% for CO_2_ splitting, respectively. The theoretical second-law thermodynamic efficiency is 55.7% and 65.7% for H_2_O splitting and CO_2_ splitting;

(3) Mechanical O_2_ separation method has a very low separation efficiency, and it limits the theoretical energy efficiency. For now, electrochemical O_2_ separation has a higher efficiency than that with mechanical separation due to its high-efficient O_2_ separation;

(4) This research provides insight into the development and potential for high-efficiency H_2_O or CO_2_ splitting, and it may guide the experimental research and further application for thermochemical solar fuel generation.

## Figures and Tables

**Table 1:** Dimensions of the OPM reactor.

*R*_o_ (cm)	*R*_in_ (cm)	*L* (cm)
0.1	0.08	80

## Data Availability

The data presented in this study are available on request from the corresponding author.
